# Protective effects of oleuropein against renal injury oxidative damage in alloxan-induced diabetic rats; a histological and biochemical study

**DOI:** 10.15171/jnp.2017.34

**Published:** 2017-02-20

**Authors:** Hassan Ahmadvand, Gholamreza Shahsavari, Majid Tavafi, Shahrokh Bagheri, Mohamad Reza Moradkhani, Reza Mohammadrezaei Kkorramabadi, Peyman Khosravi, Maryam Jafari, Khadije Zahabi, Reza Eftekhar, Maryam Soleimaninejad, Sanaz Moghadam

**Affiliations:** ^1^Razi Herbal Researches Center, Lorestan University of Medical Sciences, Khorramabad, Iran; ^2^Department of Biochemistry, Faculty of Medicine, Lorestan University of Medical Sciences, Khorramabad, Iran; ^3^Department of Anatomy, Faculty of Medicine, Lorestan University of Medical Sciences, Khorramabad, Iran; ^4^Department of Anesthesia, Madani Heart Center, Lorestan University of Medical Sciences, Khorramabad, Iran.; ^5^Student Research Committee, Lorestan University of Medical Sciences, Khorramabad, Iran.; ^6^Department of Animal Biology, Payam Noor University, Isfahan, Iran; ^7^Department of Biochemistry, Faculty of Medicine, Babol University of Medical Sciences, Babol, Iran

**Keywords:** Diabetes mellitus, Rat, Glomerulosclerosis, Oleuropein, Myeloperoxidase activity, Nitric oxide, Kidney function test

## Abstract

**Background:**

Oleuropein is a potent antioxidant and free-radical scavenger with antiinflammatory properties.

**Objectives:**

In the present study, we evaluated the protective effects of oleuropein on myeloperoxidase (MPO) activity, nitrite, urea, creatinine and glomerulosclerosis in alloxan-induced type 1 diabetic rats.

**Materials and Methods:**

Thirty Sprague-Dawley male rats were randomly divided into 3 groups: group 1 as control; group 2 as untreated diabetic; and group 3 as treated with oleuropein 15 mg/kg i.p daily. Diabetes was induced in the second and third groups by subcutaneous alloxan injection. After 48 days, the animals were anaesthetized and then the livers and kidneys were removed immediately and used fresh or kept frozen until MPO activity analysis. Blood samples were also collected before sacrificing to measure nitrite, urea, and creatinine. Kidney paraffin sections were prepared to estimate glomerular volume, leukocyte infiltration, and glomerulosclerosis.

**Results:**

Oleuropein significantly decreased leukocyte infiltration and glomerulosclerosis in the treated group compared with the diabetic untreated group. Oleuropein significantly decreased the levels of urea, nitrite, and creatinine in the treated group compared with the diabetic untreated group. Moreover, oleuropein significantly decreased MPO activity in the treated group compared with the diabetic untreated group.

**Conclusions:**

Oleuropein has antioxidative and antiatherogenic activities and exerts beneficial effects on inflammation and kidney function test and decreases diabetic complication in diabetic rats.

**Implication for health policy/practice/research/medical education:**

Our study shows oleuropein reduced the leukocyte infiltration and glomerulosclerosis and levels of nitrite, urea and creatinine and myeloperoxidase activity in diabetic treated group. The authors hope, the results of the present study help to improve kidney function and the complication in diabetic patients.

## 1. Background

Numerous studies have shown that the disturbance of oxidant–antioxidant balance system is involved in the creation and enhancement severity of various diseases such asrenal failure (1),coronary heart disease, diabetes, and many disease complications (2).Nitric oxide (NO) is an important messenger molecule in biology (3).It acts as a vasodilator, neurotransmitter, and immunological mutilators (4).It also has both antioxidative and prooxidative properties (3). NO increases in some chronic diseases such as diabetes (3).Moreover, NO has anti-inflammatory and anti-atherogenic activities (3).Myeloperoxidase (MPO) is an enzyme that is released by activated neutrophils, monocytes, and tissue-associated macrophages after inflammatory stimuli (5). Therefore, NO and MPO are good markers for the evaluation of oxidant/antioxidant, atherogenic status, and inflammation in diabetes (5).Hyperglycemia in diabetic patients directly causes the glycation of lipids, nucleic acid, and proteins, leads to cell injuries and glucose autoxidation, and induces oxidative stress and lipid peroxidation (2).Antioxidants decrease and lipid peroxidation products increase due to glycation (6).Many natural antioxidants such as coenzyme Q10 and flavonoid compounds are known to have anti-atherogenic, hypoglycemic, hypolipidemic and anti-inflammatory activities (3).The side effects of natural antioxidants are less than chemical drugs, also natural antioxidants are safe and appropriate drugs or supplements for the treatment of diabetes mellitus (2,7).

Oleuropein is a new, naturally antioxidant compound, present in large quantities in olive leaves (5,8). Oleuropein has anti-inflammatory effects by inhibiting lipoxygenase activity and producing leukotriene B4 and NO (9).Oleuropein has a high antioxidant activity in vitro, scavenges free radicals (8).It also acts as a free-radical scavenger and decreases oxidative stress, which are considered key factors in the pathogenesis of skin damage, carcinogenesis, tumor growth, aging, and bactericidal, atherosclerosis, diabetes, and neural defects (10). Oleuropein also has others properties including anti-platelet aggregation, hypotensive, anti-rheumatic, cardio-protective, and antipyretic effects (11). Since the protective effects of oleuropein on glomerulosclerosis, MPO activity, NO, and kidney function test in diabetic rats have not previously been done.

## 2. Objectives

The objectives of the present study were to investigate the protective effects of oleuropein on glomerulosclerosis, MPO activity, NO, and kidney function test in diabetic rats.

## 3. Materials and Methods

### 3. 1. Animals

Thirty male mature Sprague–Dawley rats (180-200 g) were obtained from Pasteur Institute of Tehran. The rats were divided into 3 groups (10 each): group 1 as control, group 2 as untreated diabetic, and group 3 as diabetic treated with oleuropein.

### 3.2. Diabetes induction

Diabetes was induced in the second and third groups by the subcutaneous injection of alloxan monohydrate (120 mg/kg) according to our previous study (2).The third group was treated with oleuropein 15 mg/kg i.p daily (1).The treatment began on the first day of diabetes induction. After 48 days of treatment, animals were anesthetized (Nesdonal 50 mg/kg, i.p.); blood samples were collected from hearts and allowed to clot for 20 minutes at laboratory temperature and then centrifuged at 3000 rpm for 10 minutes for serum separation (2,8,12). The kidneys were then removed immediately and used fresh or kept frozen until the analysis.

### 3.3. Histological study

The mean glomerular volume (VG), glomerulosclerosis and leukocyte infiltration were assessed according to our previous study (2).

### 3. 4. Biochemical study

#### 3. 4. 1. Levels of nitrite

Serum levels of nitrite were measured according to our previous study (13).

#### 3. 4. 2. Myeloperoxidase activity

MPO activity was determined through a modified O-dianisidine method (5).The assay mixture, in a cuvette of 1-cm path length, contained 0.3 mL of 0.1-M phosphate buffer (pH 6.0), 0.3 mL of 0.01-M H₂O₂, 0.5 mL of 0.02-M O-dianisidine (freshly prepared) in deionized water, and 10 μL of kidney or liver homogenate in a final volume of 3 mL. The kidney or liver homogenate was added in the end and the change in absorbance at 460 nm was followed for 10 minutes. All measurements were carried out in duplicate. One unit of MPO is defined as the amount which increases the absorbance of 0.001 per minute, and specific activity is defined as IU/mg protein.

#### 3. 4. 3. Levels of serum fasting blood glucose, urea, and creatinine

The serum levels of fasting blood glucose** (**FBG), urea, and creatinine were measured by a biochemical analyzer using commercial kits.

### 3.5. Ethical issues

The research followed the tenets of the Declaration of Helsinki. The research was approved by ethical committee of Lorestan University of Medical Sciences. Prior to the experiment, the protocols were confirmed to be in accordance with the Guidelines of Animal Ethics Committee of Lorestan University of Medical Sciences.

### 3. 6. Statistical analysis

All values are expressed as mean ± SEM. The data were compared between groups by Mann-Whitney U test. Statistical analyses were performed using SPSS 13 for Windows.

## 4. Results

### 4. 1. Histological results

#### 4. 1. 1. The effect of oleuropein on the glomerular volume

**Figure 1:**
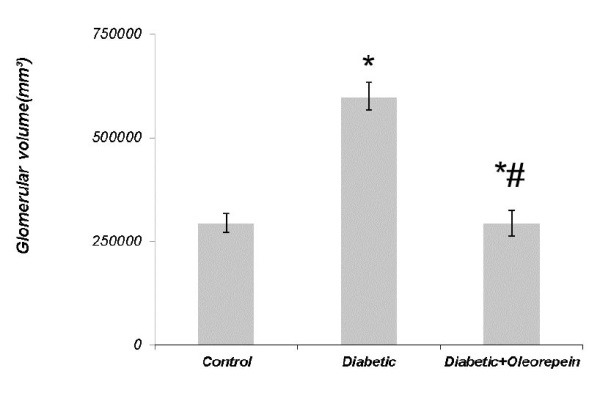
The effect of oleuropein on glomerular volume in alloxan induced diabetic rats.

The levels of glomerular volume are shown in Figure 1. The value of glomerular volume was significantly (2.04-fold) lower in control animals than untreated diabetic rats. Treatment of diabetic animals with oleuropein significantly inhibited glomerular volume (45.8%).

*P < 0.05 as compared with control group.

#*P* < 0.05 as compared with diabetic without treatment group.

#### 4. 1. 2. The effect of oleuropein on leukocyte infiltration

**Figure 2:**
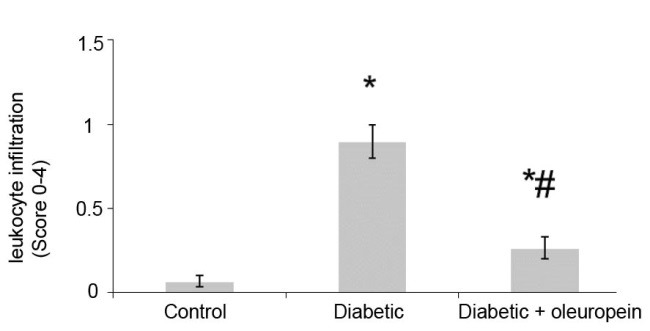
The effect of oleuropein on leukocyte infiltration in alloxan induced diabetic rats.

**Figure 3:**
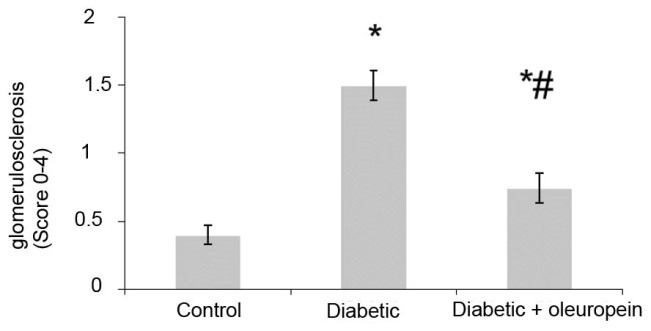
The effect of oleuropein on glomerulosclerosis in alloxan induced diabetic rats.

The levels of leukocyte infiltration are demonstrated in Figure 2. Results showed that leukocyte infiltration was significantly (13-fold) lower in control animals than the untreated diabetic group, and the level of leukocyte infiltration was significantly lower in diabetic rats treated with oleuropein than diabetic untreated animals. The treatment of diabetic animals with oleuropein significantly (70.46%) decreased leukocyte infiltration (Figure 3).

*P < 0.05 as compared with control group.

#*P* < 0.05 as compared with diabetic without treatment group.

#### 4. 1. 3. The effect of oleuropein on glomerulosclerosis

The level of glomerulosclerosis was significantly (3.74-fold) higher in the untreated diabetic rats than control animals. The treatment of diabetic animals with oleuropein significantly (50.90%) decreased glomerulosclerosis (Figure 3).

*P < 0.05 as compared with control group.

#*P* < 0.05 as compared with diabetic without treatment group.

### 4. 2. Biochemical results

#### 4. 2. 1. The effect of oleuropein on serum NO

**Figure 4:**
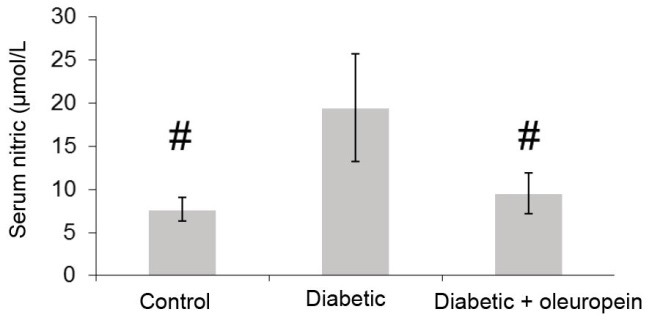
The effect of oleuropein on serum level nitric oxide in alloxan induced diabetic rats.

The serum levels of nitrite are shown in Figure 4. The level of nitrite was significantly (2.53-fold) higher in the untreated diabetic rats than control animals. The treatment of diabetic animals with oleuropein significantly (51.07%) decreased nitrite.

#*P* < .05 as compared with diabetic without treatment group.

#### 4. 2. 2. The effect of oleuropein on serum MPO activity

The MPO activities in kidneys are demonstrated in Table 1. The kidney MPO activity was significantly (2.10-fold) lower in the untreated diabetic rats than control animals. The serum MPO activity in diabetic rats treated with oleuropein was very low. The treatment of diabetic animals with oleuropein significantly (33.72%) inhibited the increase of MPO activity in comparison with the untreated diabetic animals (Table 1).

**Table 1:** The effect of oleuropein on serum, liver and renal myeloperoxidase activity in alloxan induced diabetic rats

**Groups**	**Renal MPO** **(nmol/mg protein)**	**Liver MPO** **(nmol/mg protein)**
Control	7.00±3.03	6.60±3.05
Diabetic	14.71±4.65*	14.17±8.74*
Diabetic treated	9.75±4.13#	4.70±2.11#

Values represented as mean ± SEM *P < 0.05 as compared with control group. #*P* < 0.05 as compared with diabetic without treatment group.

The MPO activities in livers are shown in Table 1. The kidney MPO activity was significantly (2.15-fold) lower in the untreated diabetic rats than control animals. The serum MPO activity in diabetic rats treated with oleuropein was very low. The treatment of diabetic animals with oleuropein significantly (66.83%) inhibited the increase of MPO activity (Table 1).

#### 4. 2. 3. The effect of oleuropein on FBG and serum kidney functional test of diabetic rats

The level of FBG was significantly (4.81-fold) higher in the untreated diabetic rats than control rats. The treatment of diabetic rats with oleuropein significantly (29%) inhibited the increase of glucose in comparison with untreated diabetic rats (Table 2). The serum levels of urea are presented in Table 2. The level of urea was significantly (4.01-fold) higher in the untreated diabetic rats than control animals. The serum level of urea in diabetic rats treated with oleuropein was low. The treatment of diabetic animals with oleuropein significantly (50.52%) decreased urea in comparison with untreated diabetic animals. The level of creatinine was significantly (4.44-fold) higher in the untreated diabetic rats than control animals. The treatment of diabetic animals with oleuropein slightly decreased creatinine (Table 2).

**Table 2:** The effect of oleuropein on serum urea and creatinine in alloxan induced diabetic rats

**Groups**	**FBG** **(mg/dL)**	**Serum Urea (mg/dL)**	**Serum creatinine** **(mg/dL)**
Control	106.45 ± 18.44	43.83±11.55	0.61±0.17
Diabetic	463.00±178.02^*^	175.43±65.43^*^	2.71±0.72^*^
Diabetic treated	294.40±54.63^*#^	86.80±21.22^*#^	2.45±1.64_*_

Values represented as mean ± SEM *P < 0.05 as compared with control group. #*P* < 0.05 as compared with diabetic without treatment group.

## 5. Discussion

This study showed that oleuropein has beneficial effects in decreasing the elevated serum NO, MPO, urea, and creatinine, and protective effects on leukocyte infiltration and glomerulosclerosis in diabetic rats. Oxidative stress plays a key role in most pathogenic pathways of diabetic injuries, inflammation, carcinogenesis (13,14). Antioxidants such as vitamin E, alpha-lipoic acid, and oleuropein protect the cells against oxidative stress (15,16).Therefore, the use of antioxidants as a complementary therapy is useful in oxidative stress-related diseases.

### 5. 1. Histological studies

The glomerular volume, glomerulosclerosis and leukocyte infiltration significantly increased in untreated diabetic animals. Oleuropein significantly decreased glomerular volume, glomerulosclerosis and leukocyte infiltration in treated diabetic animals. Several studies showed that vitamin E, coenzyme Q10, vitamin C, alpha-lipoic acid and rosmarinic acid, and vitamin E can decrease nephropathic defects in diabetic rats (15-17).The histological evaluation of heart and liver indicated that oleuropein reduced inflammation and fibrosis in the induced model of metabolic syndrome (18).Other ex vivo studies showed that oleuropein brought about a marked improvement in vascular function in the aortic rings derived from treated rats (19).Our previous study indicated that coenzyme Q10 and rosmarinic acid reduce nephropathic defects in diabetic nephropathy rats (2).Also our study showed that oleuropein reduces nephropathic defects in diabetic rats. Therefore, oleuropein with beneficial effects on nephropathic defects can be helpful in reducing the complications related to inflammation, vascular health and oxidative stress in diabetic patients.

### 5. 2. Biochemical studies

#### 5. 2. 1. The effect of oleuropein on serum nitrite level and MPO activity

The level of serum nitrite and MPO activity significantly increased in untreated diabetic animals. Oleuropein significantly decreased serum nitrite and MPO activity in treated diabetic animals.

NO is a good marker for the evaluation of endothelial function,nervous system health,immune systems,and oxidative stress status (3).Moreover, NO has anti-inflammatory, anti-ulcer, and anti-atherogenic activities, increases insulin sensitivity, protects blood vessels from endogenous injury, and plays a key role in metabolic and cardiovascular homeostasis (3). Natural antioxidants such as black and green tea,epigallocatechin gallate (20),quercetin, and vitamin E (21) decrease the circulation of NO with protective effects on blood pressure, vascular health, endothelial function, inflammation, and insulin sensitivity. Several studies showed that natural antioxidants such as α-carbolines, α-tocopherol (22),quercetin, and coenzyme Q10 (23) improve NO in vivo. In addition, MPO is an appropriate marker for the evaluation of oxidant/antioxidant, inflammatory, and atherogenic status (5).Based on reports, natural antioxidants such as atorvastatin,quercetin, and deprenylimprove MPO activity in vivo (24). Results of our study and other studies showed that oleuropein decreased serum NO level and MPO activity. Therefore, oleuropein with beneficial effects on NO and MPO activity can be helpful in reducing the complications related to inflammation, vascular health, oxidative stress and endothelial function in diabetic patients.

#### 5. 2. 2. The effect of oleuropein on serum urea and creatinine

Serum urea and creatinine levels as markers of renal function test significantly increased in diabetic animals in comparison with the control group. Oleuropein significantly decreased serum urea and slightly decreased creatinine level in treated diabetic animals in comparison with the untreated diabetic animals. Several studies showed that natural antioxidants such as selenium, vitamin E, vitamin C (25), and alpha-lipoic acid (15) decrease serum urea and creatinine. Our previous study demonstrated that coenzyme Q10 and rosmarinic acid decrease serum urea and creatinine in diabetic rats (2). There Based on reports, oleuropein has beneficial effects LDL oxidation and lipid peroxidation (9). It also has anti-inflammatory effects.The results of our study are in accordance with those of other studies showing that oleuropein can decrease NO, urea, and creatinine levels and MPO activity. Oleuropein can also decrease renal injury such as glomerulosclerosis. Therefore, natural antioxidants can improve various tissue damages observed in diabetic patients (9).

Natural antioxidant therapy is one of the best treatment strategies in chronic diseases such as diabetes to prevent and slow the progression of diseases and their complications such as insulin sensitivity defect, vascular health damage, inflammation, and hepatic and kidney damage. As a result, oleuropein as a natural good antioxidant can inhibit the progression of diabetic complications.

## 6. Conclusions

This study indicated that oleuropein decreases elevated serum NO and kidney and liver MPO activity, and has beneficial effects on kidney function test, leukocyte infiltration, glomerular hypertrophy and glomerulosclerosis in diabetic rats. These ameliorative properties can decrease diabetic complications such as inflammation and atherogenic process chronic diseases.

## Acknowledgments

The authors wish to thank Deputy of Research and Razi Herbal Research Center of Lorestan Medical University, Lorestan, Iran.

## Authors’ contribution

HA, GS and MRM designed the project. RE, MSN and SM collected the data. TS analyzed the data. SB, MRM, RMK, PK, MJ and KZ; wrote the manuscript. HA, GS; revised English version. HA edited the final draft. All authors signed the manuscript.

## Conflicts of interest

The authors declare no conflict of interest.

## Funding/Support

This research was supported by Lorestan University of Medical Sciences (Grant# 21/91).

## References

[1] Ahmadvand H, Ghasemi Dehnoo M, Dehghani A, Bagheri S, Cheraghi RA (2014). Serum paraoxonase 1 status and its association with atherogenic indexes in gentamicin-induced nephrotoxicity in rats treated with coenzyme Q10. Ren Fail.

[2] Ahmadvand H, Tavafi M, Khosrowbeygi A (2012). Amelioration of altered antioxidant enzymes activity and glomerulosclerosis by coenzyme Q10 in alloxan-induced diabetic rats. J Diabetes Complications.

[3] Murad F (2004). Discovery of some of the biological effects of nitric oxide and its role in cell signaling. Biosci Rep.

[4] Bakhtiari N, Hosseinkhani S, Larijani B, Mohajeri-Tehrani MR, Fallah A (2012). Red blood cell ATP/ADP &amp; nitric oxide: the best vasodilators in diabetic patients. J Diabetes Metab Disord.

[5] Khalatbary AR, Ahmadvand H (2012). Short report effect of oleuropein on tissue myeloperoxidase activity in experimental spinal cord trauma. Iran Biomed J.

[6] Al-Musayeib N, Perveen S, Fatima I, Nasir M, Hussain A (2011). Antioxidant, anti-glycation and anti-inflammatory activities of phenolic constituents from Cordia sinensis. Molecules.

[7] Khosla P, Daud ZA, Tubie B, Sheyman M, Osia R, Adams J (2013). Vitamin E tocotrienol supplementation improves lipid profiles in chronic hemodialysis patients. Vasc Health Risk Manag.

[8] Pourkhodadad S, Alirezaei M, Moghaddasi M, Ahmadvand H, Karami M, Delfan B (2016). Neuroprotective effects of oleuropein against cognitive dysfunction induced by colchicine in hippocampal CA1 area in rats. J Physiol Sci.

[9] Khalatbary AR, Zarrinjoei GR (2012). Anti-inflammatory effect of oleuropein in experimental rat spinal cord trauma. Iran Red Crescent Med J.

[10] Sarbishegi M, Mehraein F, Soleimani M (2014). Antioxidant role of oleuropein on midbrain and dopaminergic neurons of substantia nigra in aged rats. Iran Biomed J.

[11] Omar SH (2010). Oleuropein in olive and its pharmacological effects. Sci Pharm.

[12] Shahsavari R, Ehsani-Zonouz A, Houshmand M, Ahangari G, Firoozra M (2009). Plasma glucose lowering effect of the wild Satureja khuzestanica jamzad essential oil in diabetic rats: role of decreased gluconeogenesis. Pak J Biol Sci.

[13] Petrola MJ, de Castro AJ, Pitombeira MH, Barbosa MC, Quixadá AT, Duarte FB (2012). Serum concentrations of nitrite and malondialdehyde as markers of oxidative stress in chronic myeloid leukemia patients treated with tyrosine kinase inhibitors. Rev Bras Hematol Hemoter.

[14] Selvaraju V, Joshi M, Suresh S, Sanchez JA, Maulik N, Maulik G (2012). Diabetes, oxidative stress, molecular mechanism, and cardiovascular disease--an overview. Toxicol Mech Methods.

[15] Ahmadi A, Mazooji N, Roozbeh J, Mazloom Z, Hasanzade J (2013). Effect of alpha-lipoic acid and vitamin E supplementation on oxidative stress, inflammation, and malnutrition in hemodialysis patients. Iran J Kidney Dis.

[16] Jemai H, El Feki A, Sayadi S (2009). Antidiabetic and antioxidant effects of hydroxytyrosol and oleuropein from olive leaves in alloxan-diabetic rats. J Agric Food Chem.

[17] Tavafi M, Ahmadvand H (2011). Effect of rosmarinic acid on inhibition of gentamicin induced nephrotoxicity in rats. Tissue Cell.

[18] Puel C, Mathey J, Agalias A, Kati-Coulibaly S, Mardon J, Obled C (2006). Dose-response study of effect of oleuropein, an olive oil polyphenol, in an ovariectomy/inflammation experimental model of bone loss in the rat. Clin Nutr.

[19] Bali EB, Ergin V, Rackova L, Bayraktar O, Küçükboyaci N, Karasu Ç (2014). Olive leaf extracts protect cardiomyocytes against 4-hydroxynonenal-induced toxicity in vitro: comparison with oleuropein, hydroxytyrosol, and quercetin. Planta Med.

[20] Kumar P, Kumar A (2009). Protective effects of epigallocatechin gallate following 3-nitropropionic acid-induced brain damage: possible nitric oxide mechanisms. Psychopharmacology (Berl).

[21] Qureshi AA, Reis JC, Qureshi N, Papasian CJ, Morrison DC, Schaefer DM (2011). δ-Tocotrienol and quercetin reduce serum levels of nitric oxide and lipid parameters in female chickens. Lipids Health Dis.

[22] Zal F, Mostafavi-Pour Z, Amini F, Heidari A (2012). Effect of vitamin E and C supplements on lipid peroxidation and GSH-dependent antioxidant enzyme status in the blood of women consuming oral contraceptives. Contraception.

[23] Coldiron AD Jr, Sanders RA, Watkins JB (2002). Effects of combined quercetin and coenzyme Q10 treatment on oxidative stress in normal and diabetic rats. J Biochem Mol Toxicol.

[24] Wang P, Vadgama JV, Said JW, Magyar CE, Doan N, Heber D (2014). Enhanced inhibition of prostate cancer xenograft tumor growth by combining quercetinand green tea. J Nutr Biochem.

[25] Karabulut-Bulan O, Bolkent S, Yanardag R, Bilgin-Sokmen B (2008). The role of vitamin C, vitamin E, and selenium on cadmium-induced renal toxicity of rats. Drug Chem Toxicol.

